# *Hansenia weberbaueriana* (Fedde ex H.Wolff) Pimenov & Kljuykov Extract Suppresses Proliferation of HepG2 Cells via the PTEN-PI3K-AKT Pathway Uncovered by Integrating Network Pharmacology and *Iin Vitro* Experiments

**DOI:** 10.3389/fphar.2021.620897

**Published:** 2021-04-20

**Authors:** Yueqin Feng, Fengjin Hao

**Affiliations:** ^1^ Department of Ultrasound, The First Affiliated Hospital of China Medical University, Shenyang, China; ^2^ Department of Biochemistry and Molecular Biology, China Medical University, Shenyang, China

**Keywords:** Hansenia weberbaueriana, network pharmacology, HepG2 cell, apoptosis, PTEN-PI3K-akt pathway

## Abstract

Previous studies have shown that *Hansenia weberbaueriana* (Fedde ex H.Wolff) Pimenov & Kljuykov extracts (HWEs) have antitumor activity, but their mechanism *in vitro* is still unclear. In this study, we first combined network pharmacology with experimental evaluation and applied a comprehensive strategy to explore and prove the therapeutic potential and potential mechanism of HWE. The mRNA expression profiles of PTEN, PIK3A, and AKT1 are from the Cancer Cell Line Encyclopedia (CCLE) of the Broad Institute. Our results showed that HWE has a good inhibition on HepG2 cells, and a slight inhibition on other cells. The results of the CCLE database showed that PTEN/PIK3A/AKT1 mRNA expression was up-regulated in HepG2 cells. Through further study, it was found that HWE increased the release of LDH, induced early and late apoptosis, and increased ROS levels in HepG2 cells. Western blot showed that HWE regulates the expression of mitochondrial apoptosis-related proteins. Meanwhile, the expression of PTEN was increased, and the expression of phosphorylated PI3K and Akt was down-regulated after HWE treatment. Our results show that HWE promotes HepG2 cell apoptosis via the PTEN-PI3K-Akt signaling pathway. This study is the first to report the potential role of HWE in the treatment of liver cancer.

## Introduction

Liver cancer is one of the major causes of cancer-related mortality all over the world, with hepatocellular carcinoma (HCC) accounts for 95% of liver cancers (Hartke et al., 2017). At present, the most effective treatment for patients with HCC is surgical resection. Progressive HCC is rarely treated radically by surgery or *in situ* liver transplantation (Abou-Alfa et al., 2006), and traditional chemotherapeutic agents are multi-drug resistant and have a high incidence of toxic side effects, which seriously affects the quality of life of patients. This is essential for maintaining normal physiology, but the overall recurrence rate is 50–60% (Kudo 2011; Ang et al., 2015; Ma and Cheung, 2017), Therefore, further research on the molecular mechanisms of HCC tumorigenesis is essential for coping with life-threatening HCC.

Traditional Chinese Medicine (TCM) has been used in Asia for more than 2,000 years. As one of the most popular supplements and alternative medicines in China, TCM is gradually accepted by non-Chinese medicine due to its outstanding efficacy, abundant resources, and low toxicity (Guo et al., 2019). It is believed that TCM is characteristic of its multi-component and multi-target. The complexity of the herbal mixture and limited understanding of its molecular mechanism limit the use and development of TCM (Huang et al., 2020). Network pharmacology is a new and powerful tool that helps us understand the complex interactions between target proteins and small molecules from a biological perspective, similar to the holism concept of channel tropism in TCM (Wang et al., 2017).

*Hansenia weberbaueriana* (Fedde ex H.Wolff) Pimenov & Kljuykov (syn. *Notopterygium incisum* K. C. Ting ex H. T. Chang) ex H.T. Chang (*N. incisum*) is TCM in China and is mainly distributed in the northwest and southwest of China. The main chemical components of *H. weberbaueriana* are coumarin, organic acids, volatile oils, amino acids, and other compounds (Azietaku et al., 2017). The pharmacological effects of HWE have been widely reported in recent years, including antiviral, anti-inflammatory, antioxidant, and anti-Alzheimer’s disease properties (Jiang et al., 2020). Previously, it was reported that chloroform extract of *H. weberbaueriana* inhibited PSN-1 and PANC-1 cells (Azietaku, et al., 2017). In the current research, we use computational tools and resources to study the pharmacological network of HWE to predict active compounds and potential protein targets and pathways. In addition, the potential mechanism of HWE was verified *in vitro*.

## Materials and Methods

### Network Pharmacology-Based Analysis

#### Identification of Candidate Components in *Hansenia weberbaueriana*

All phytochemicals of the *H. weberbaueriana* were retrieved from Traditional Chinese Medicine on Immuno-Oncology (TCMIO) database (http://tcmio.xielab.net/) (Liu et al., 2020).

#### Prediction of Drug Targets for *Hansenia weberbaueriana*

The protein targets of the active substances in *H. weberbaueriana* were retrieved from the SwissTargetPrediction (http://www.swisstargetprediction.ch) (Daina et al., 2019) and TCMIO database.

#### Gene Ontology and Pathway Enrichment Analysis

The gene ontology (GO) and pathway enrichment analyses were conducted using NetworkAnalyst 3.0 (Zhou et al., 2019). All genes in the genome have been used as the enrichment background. We collected the *p* value < 0.01, the minimum count as 3 and the enrichment factor >1.5, and we grouped them according to the similarity of their members. When performing hierarchical clustering on rich terms, a Kappa score of 4 was used as the similarity metric, and a subtree with similarity >0.3 was regarded as a cluster. We chose the most statistically significant term in the cluster to represent the cluster.

#### Construction of Networks and Analysis

In order to further explore the relationship between paths, a subset of rich terms was selected and presented as a network graph in which a similarity >0.3 was connected by edges.

#### Protein–Protein Interaction Enrichment Analysis

We used the Search Tool for the Retrieval of Interacting Genes (STRING, https://string-db.org/) as previously described (Jiang et al., 2018). The resulting network contains a subset of proteins that form a physical interaction with at least one other member of the list. If the network contains 3*–*500 proteins, the MCODE scores >3 and the number of nodes >4 were used as cut-off criteria. A value *p* < 0.05 was considered to indicate significant differences.

#### *PTEN/PIK3A/AKT1* mRNA Expression

The *PTEN/PIK3A/AKT1* mRNA expression of 100 human cancer cell lines was obtained from the Broad Institute Cancer Cell Line Encyclopedia (CCLE).

### Experimental Validation

#### Preparation of HWE

*Hansenia weberbaueriana* was purchased from Shenyang Pharmacy in June 2019. It was identified by Professor Wang Lannian of Liaoning University of Traditional Chinese Medicine. Specimens (voucher No. Lian201906) were kept in the People’s Hospital of China Medical University.

The dry *N. incisum* root (0.5 kg) was crushed and extracted with 95% ethanol (2L × 3) three times and for 2 h each time. After refluxing and drying, HWE was obtained and stored at 4°C.

#### Cell Culture

The HepG2, MCF-7, A549, SW480, and HeLa cells were obtained from Cyagen Biosciences Inc. (Guangzhou, China) and cultured in complete DMEM (Hycolone, United States) medium containing 10% (v/v) FBS (Clark, United States) and 1% penicillin/streptomycin (Solarbio, China). The cell incubator was set as follows: 5% CO_2_ and 37°C.

#### MTT Assay

HepG2, MCF-7, A549, SW480, and HeLa cells were incubated in 96-well plates. In order to calculate IC_50_, cells were treated with different concentrations of HWE (6.25, 12.5, 25, 50, and 100 μg/ml) after 24 h of growth. In order to further study the anti HepG2 cell proliferation activity of HWE, the concentrations of HWE were set at 10, 20, and 40 μg/ml in the following experiments Six duplicate wells of each concentration were set up with blank control wells. After 24 h of incubation, MTT (Sigma, United States) solution (final concentration at 5 mg/ml) was added to 96-well plates, and they continued to incubate in the cell incubator for 4 h before being removed. After discarding the supernatant, we added 150 μL of DMSO. Furthermore, we investigated the effect of HWE on the cell viability of normal hepatocytes HL-7702 cells, and the method was the same as above.

#### LDH Assay

Under normal conditions, LDH cannot be released through the plasma membrane. When the target cell is damaged by an attack, LDH will be released after the change of plasma membrane permeability (Jurisic et al., 2015). The final concentrations of HWE were 10, 20, and 40 μg/ml. After incubation for 24 or 48 h, LDH release in HepG2 and MCF-7 cells was measured according to the instructions of the LDH kit (Beyotime Biotechnology, China). After the reaction, the OD value was measured at 450 nm. The experiment was repeated three times in parallel. We determined the corresponding absorbance value of LDH enzyme standard with known concentrations and calculated the LDH enzyme activity in the sample.

#### Colony Formation

The cells were seeded on a 6-well plate, cultured overnight, and treated with different concentrations of DMSO or HWE for 48 h. It was then washed with PBS and cultured in complete growth medium for another 10 days. We changed the fresh medium every 3 days. After fixing with 100% methanol, the cells were stained with 0.1% crystal violet.

#### Hoechst Staining

HepG2 cells were incubated in 6-well plate. Then added different concentrations of HWE (10, 20, and 40 μg/ml) into each well and incubated for 48 h. We discarded the medium and replaced it with a fresh serum-free medium. We added 1 μL 5 mg/ml Hoechst 33,342 dye (sigma, United States) solution to each well and continued the incubation for 30 min. After incubation, the medium containing dye was discarded, washed two times with precooled PBS, and photographed by fluorescence microscope.

#### Annexin V-FITC/PI Staining

After treated with HWE for 48 h, the HepG2 cells were fully digested by trypsin to obtain cell suspension. Cells were centrifuged at 4°C and 3,000 rpm for 10 min, then they were fully resuspended and washed. Then the cells were centrifuged at 4°C and 2,000 rpm for 10 min. We discarded the supernatant, added the binding buffer, annexin V-FITC, and PI working solution (BD pharmingen, United States) to each sample, mixed them evenly, and then filtered them with a 400 mesh filter to obtain single-cell suspension. The number of apoptotic cells was measured by flow cytometry.

#### ROS Assay

The intracellular ROS production was measured by a DCFH-DA detection kit (Beyotime Biotechnology, China). The HepG2 cells were incubated with or without NIE for 6 h, incubated in the dark at 37°C, and kept away from light for 20 min with 10 μM DCFH-DA. PTEN inhibitor (Oroxin B) was used as the positive control. The cells were washed and then detected with a fluorescence microscope.

#### Western Blot

The HepG2 cells were harvested, washed, and centrifuged. Then added to 50 μL of RIPA lysis buffer and put on ice for 30 min. Then, centrifuged at 1.2 × 10^4^ rpm for 10 min, and the protein concentration of the supernatant was measured using the BCA method (Beyotime, China). We then boiled the protein sample for 10 min, and the protein sample (20 μg) was loaded onto 10% SDS-PEGE (Beyotime, China) and separated. Primary antibodies were: Bax (Wanleibio, 1:1,500), Bcl2 (Wanleibio, 1:1500), Bad (Wanleibio, 1:1,500), caspase-3 (Wanleibio, 1:1,500), caspse-9 (Wanleibio, 1:1,500), cytochrome C (Wanleibio, 1:1500), PTEN (Cell Signaling Technology, 1:1,000), p-PI3K (Cell Signaling Technology, 1:1000), PI3K (Cell Signaling Technology, 1:1,000), AKT (Cell Signaling Technology, 1:1,000), and *p*-AKT (Cell Signaling Technology, 1:1,000) were incubated at 4°C for 12 h *β*-actin (Wanleibio, 1:15,000). The gray value of the Western blot result was detected through ImageJ software.

### Statistical Analysis

All data were represented by mean ± SD. Significant differences were calculated in GraphPad 5.0 (Inc. La Jolla, CA, United States) with one-way ANOVA analysis, and then Student t-test or Tukey’s multiple comparison test was conducted. A value *p* < 0.05 indicated a significant difference.

## Results

### Identification of the Potential Bioactive Compounds of Hansenia weberbaueriana and Target Identification

**FIGURE 1:**
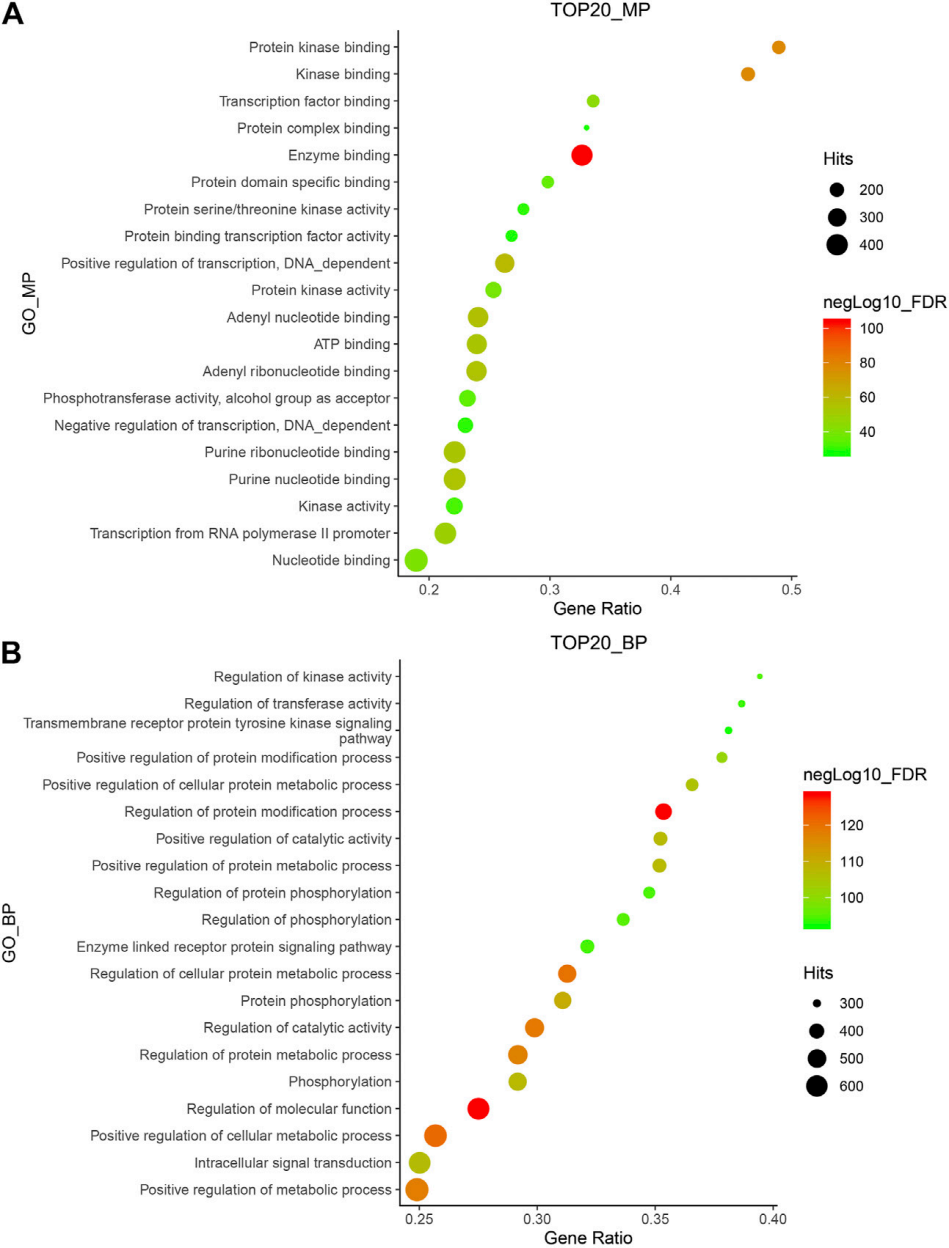
GO_MP **(A)** and GO_BP **(B)** enrichment of *Hansenia weberbaueriana*. The *x*-axis displays as gene ratio (Hits/Total) and the *y*-axis represents the GO_MP/B*p* terms. The intensity of the color depends on the −log10(FDR) and red means a higher significant difference. The size of the point shows the number of Hits.

A total of 154 compounds were retrieved from *Hansenia weberbaueriana*, see Supplementary Material for details. A total of 812 protein targets were retrieved from SwissTargetPrediction, the detailed information was shown in Supplementary Material. We analyzed the top 20 MP entries. As shown in Figure 1A, the enrichment of enzyme binding was the most significant, hitting 392 items. Among the hit 121 GO_BP items, regulation of molecular function and regulation of protein modification process are very significant (Figure 1B).

### Pathway enrichment and Networks Analysis

**FIGURE 2:**
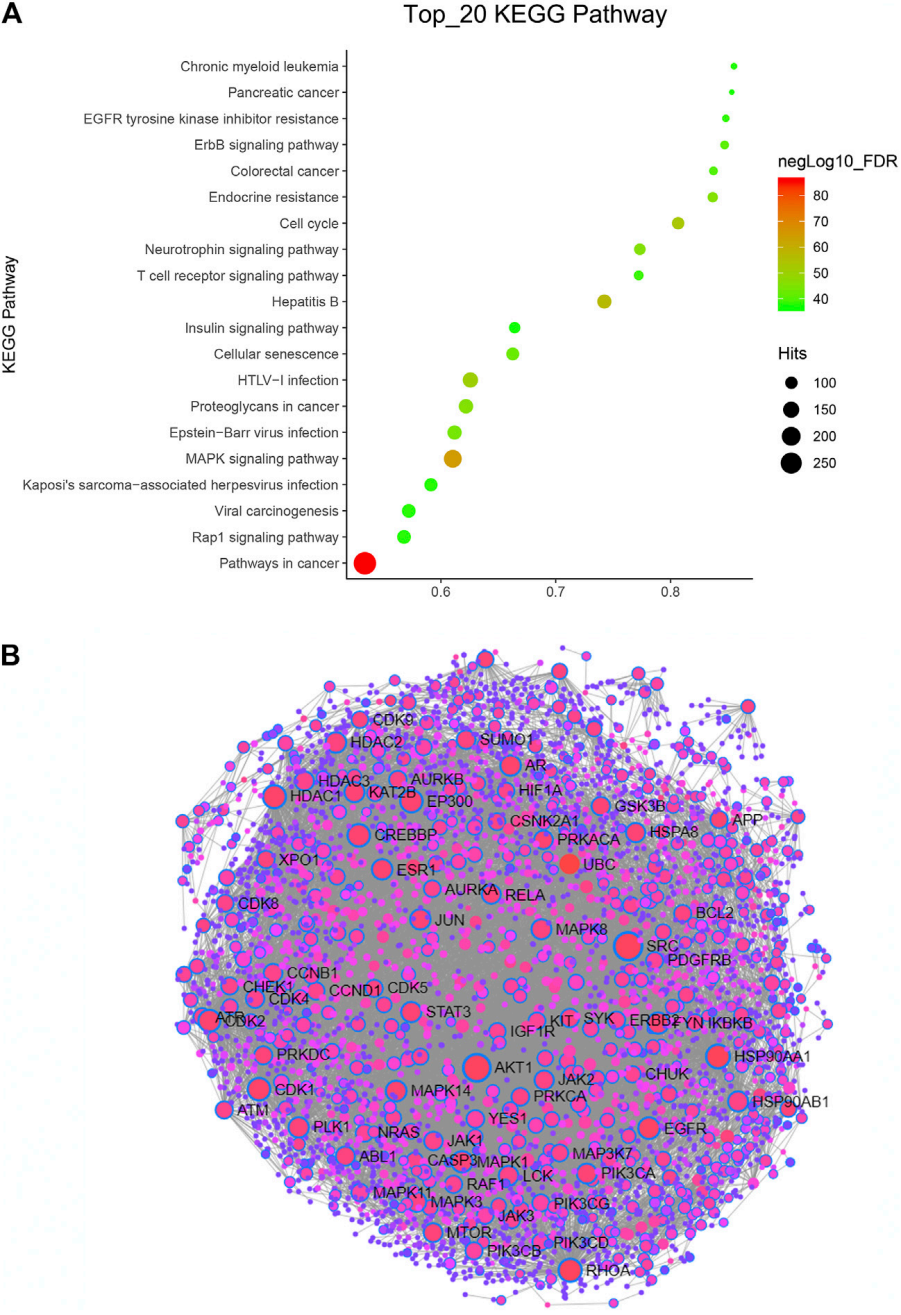
KEGG pathway and PPI network analysis of *Hansenia weberbaueriana*. **(A)** KEGG pathway analysis. The *x*-axis displays as gene ratio (Hits/Total) and the *y*-axis represents the GO_MP/BP terms. The intensity of the color depends on the −log10(FDR) and red means a higher significant difference. The size of the point shows the number of Hits. **(B)** PPI analysis. The circle represents the target gene, the line represents the interaction, and the gene with the name represents the highly related target.

Kyoto Encyclopedia of Genes and Genomes (KEGG) pathway enrichment analysis was performed using the functional annotation tool of NetworkAnalyst 3.0. We analyzed the top 20 pathways and found that pathways in cancer were very significant, as they hit 283 targets. In addition, the FDR of the MAPK signaling pathway was also very significant (Figure 2A). These two pathways are classic tumor pathways, indicating that *Hansenia weberbaueriana* has a potential anticancer effect.

In order to find the most relevant key targets, we further performed protein-protein interaction (PPI) Enrichment Analysis. As showed in Figure 2B, of the 89 nodes, we focused on the top two hub genes: the degree of SRC was 218, and the degree of SRC was 182. These two targets are the hot targets of cancer research.

**FIGURE 3:**
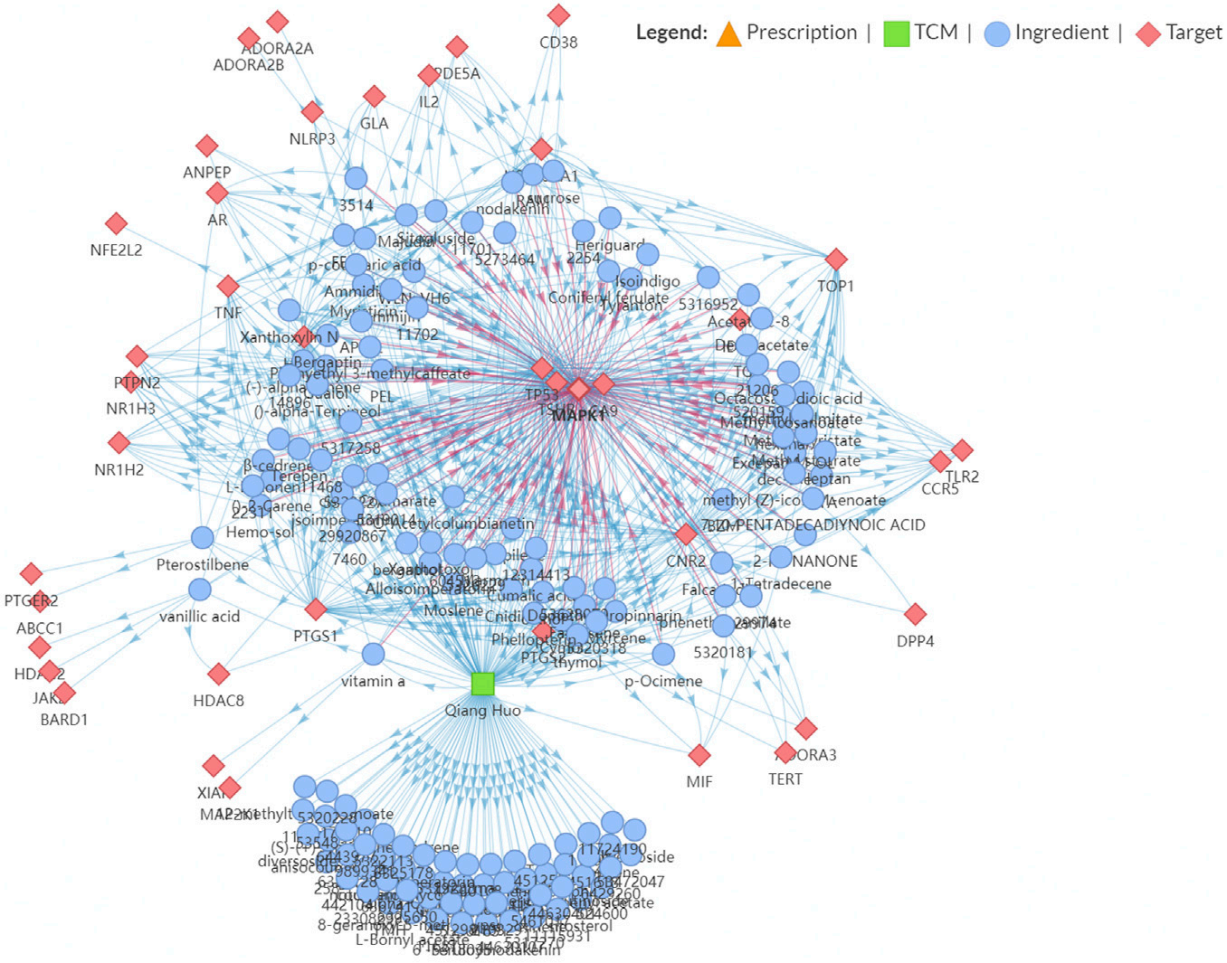
Snapshot of Hansenia weberbaueriana–ingredient–target network related to Immuno-Oncology (IO).

We further performed an immuno-oncology (IO) network analysis using the TCMIO database. As illustrated in Figure 3, the enrichment fractions of four hub targets are very significant, which are MAPK1, TP53, CA9, and TSHR.

### The mRNA Levels of PTEN/PIK3A/AKT1 in Different Tumor Cells

**FIGURE 4:**
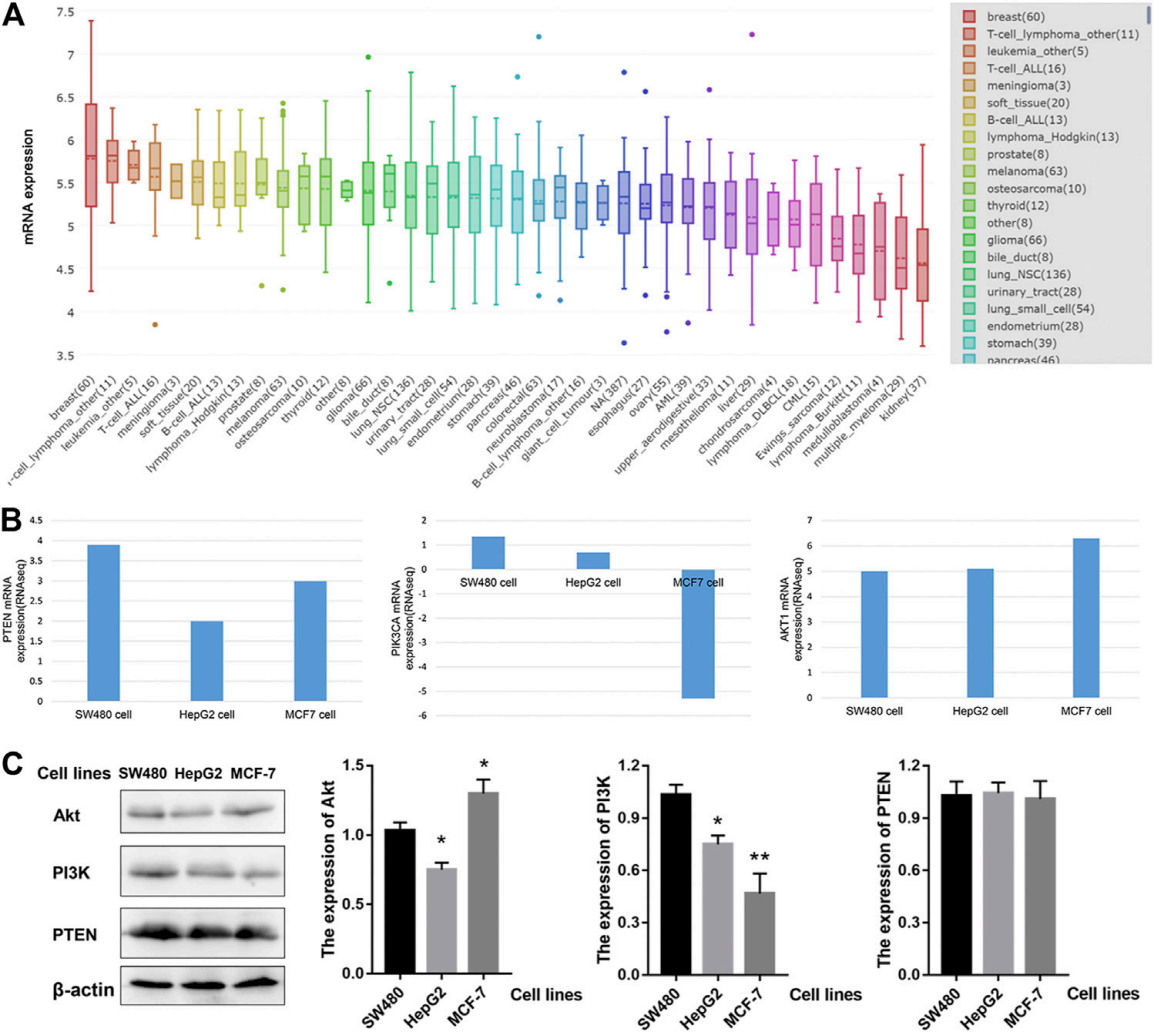
The expression of PTEN/PIK3A/Akt mRNA level among cancer cell lines. **(A)** AKT1 mRNA level in different cell lines. **(B)** The expression of PTEN/PIK3A/Akt in SW480, HepG2, and MCF-7 cells. **(C)** The protein expression levels of PI3K-Akt-PTEN in SW480, HepG2, and MCF-7 cell lines. *p < 0.05, **p < 0.01 vs control group.

Next, we investigated the mRNA levels of *PTEN*/*PIK3A*/*AKT1* in different tumor cells. As shown in Figure 4A, *AKT1* is expressed differently in different tumor cells. The highest expression of *AKT1* was found in breast cancer cell lines, with a median of 5.8 among 60 cell lines, but the heterogeneity was also the largest. Based on previous informatics data, we believe that PTEN-PI3K-AKT pathway may be involved in the anti-tumor effect of HWE. Therefore, we used the CCLE database to analyze *PTEN/PIK3A/Akt* mRNA expression in different tumor cells. Since HeLa and K562 cells were not included in the CCLE database, we analyzed SW480, MCF-7, and HepG2 cells. As shown in Figure 4B, *PTEN* and *AKT1* were highly expressed in SW480, MCF-7, and HepG2 cells. Interestingly, the expression level of PIK3A in these three cell lines was not high, especially since the expression of MCF was down-regulated. Combined with the experimental data of MTT, we analyzed the effect of HWE on the PTEN-PI3K-AKT pathway of HepG2 cells and found that it may be more significant. Furthermore, we tested the protein expression levels of PI3K-Akt-PTEN in SW480, HepG2, and MCF-7 cell lines. As shown in Figure 4C, the protein expression level of PI3K-Akt-PTEN is consistent with the mRNA expression level in the database.

### HWE Suppressed the Proliferation of Human Cancer Cells

**FIGURE 5:**
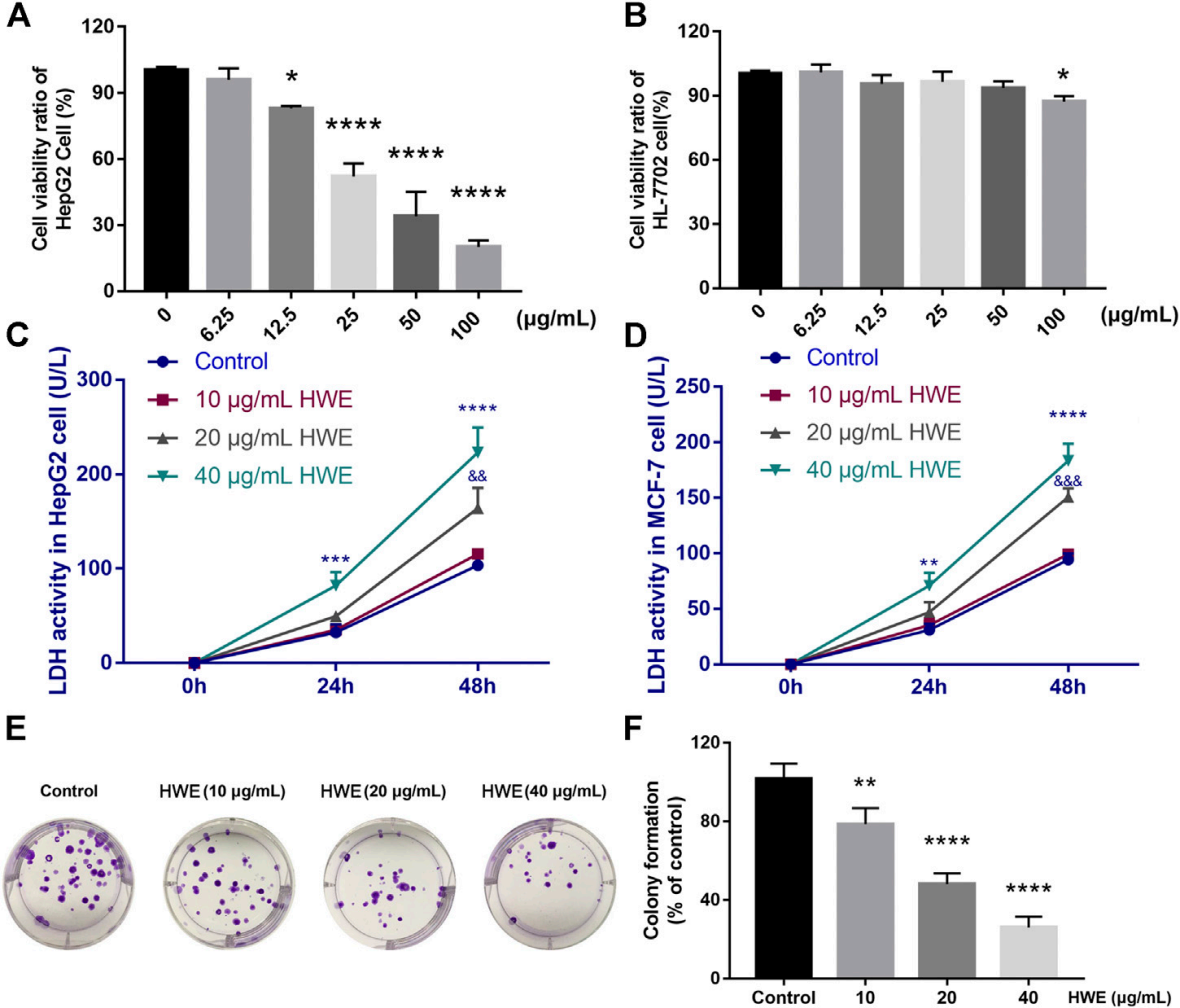
Effect of HWE on HepG2 cell viability and LDH release. **(A)** Cell viability of HepG2 cells with or without HWE treated **(B)** Cell viability of HL-7702 cells with or without HWE treated. **(C,D)** LDH activity in HepG2 and MCF-7 cells with or without HWE treated at 24 and 48 h. **(E,F)** Growth inhibition effects of HWE on HepG2 cells were measured by colony formation assay. Error bars represent SD. *p < 0.05, **p < 0.01, ****p < 0.0001 vs. control group.

An MTT assay was performed to detect the effect of HWE on the cell viability of human cancer cells. Table 1 showed that HWE could significantly inhibit the cytotoxicity of HepG2, Hep3B, SW480, MCF-7, K562, and proliferation of Hela cells. The IC_50_ value of the HWE on HepG2 cells was 22.09 ± 2.31 μg/mL. As shown in Figure 5A, HWE inhibited HepG2 proliferation in a concentration-dependent manner. Moreover, we further investigated the effect of HWE on the cell viability of normal hepatocytes HL-7702 cells. As shown in Figure 5B, the survival of HL-7702 cells was significantly inhibited when the HWE concentration was 100 μg/ml.

**TABLE 1:** Cell viability of HWE on human cancer cells.

Cell lines	IC_50_ (μg/ml)
MCF-7	38.92 ± 1.02
SW480	42.38 ± 2.93
Hela	103.23 ± 1.92
HepG2	22.09 ± 2.31
K562	98.24 ± 1.11

### HWE Induced LDH Release From HepG2 Cells

The LDH level in HepG2 cells was increased by NIE in a concentration-dependent manner. Compared with the control group, HWE (10, 20, and 40 μg/ml) caused a significant increase in LDH release within 48 h (Figure 5C). Similarly, we also found that HWE could significantly promote the release of LDH in MCF-7 cells, but it was slightly less than that in HepG2 cells (Figure 5D).

### HWE Suppressed the Colony Formation of HepG2 Cells

The long-term efficacy of NIE toward HepG2 cells was assessed by a colony formation assay. Figures 4, 5E,F showed that the HWE -treated HepG2 cells lost the capacity to proliferate in a concentration-dependent manner. The results of the colony formation assay are consistent with the growth inhibitory activity indicated by the MTT assay.

### HWE Could Induce Apoptosis in HepG2 Cells

**FIGURE 6:**
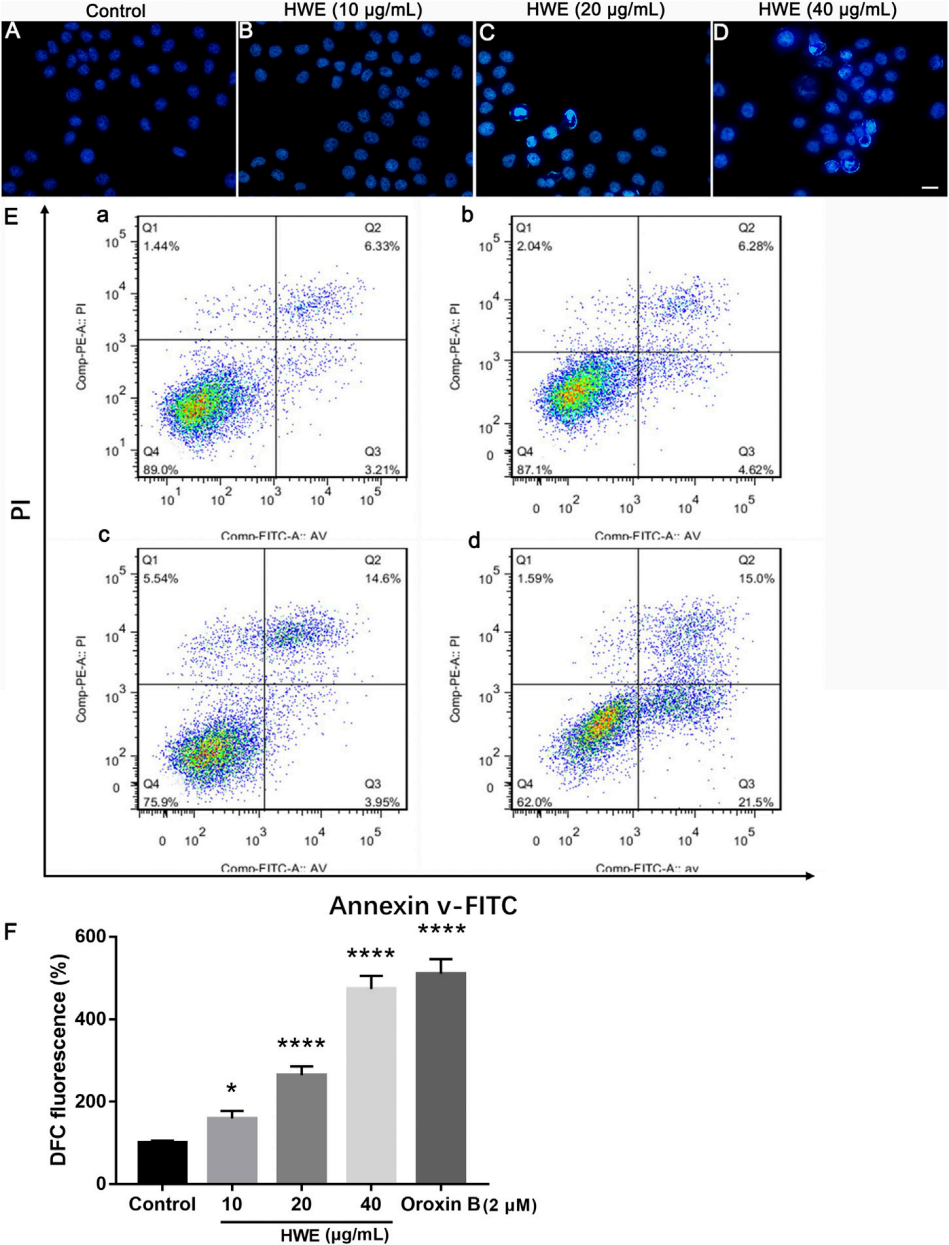
Effects of HWE on HepG2 cells cell morphology and apoptosis. **(A–D)** Hoechst 33,342 staining of HepG2 cells with or without HWE treated. **(A)** control group; **(B)**, 10 μg/ml HWE treated group; **(C)** 20 μg/ml HWE treated group; **(D)** 40 μg/ml HWE treated group. Scale bar: 20 μm. **(E)** FACS analysis of Annexin V/PI staining in HepG2 cells with or without HWE treated. **(F)** Quantitative analysis of FACS. The error bars denote SD. *p < 0.05 and **p < 0.01 vs control group.

As shown in Figures 6A–D, after Hoechst 33,342 staining, HepG2 cells showed normal nuclear morphology and even distribution of chromatin in the nucleus. However, after 48 h of treatment with different concentrations of HWE, HepG2 cells showed morphological characteristics such as chromatin agglutination and apoptosis (bright fluorescence). The results revealed that HWE induced apoptosis of HepG2 cells.

We performed the Annexin-V-FITC/PI staining to observe the effect of HWE on apoptosis of HepG2 cells. Figures 6E,F showed that HWE induced early and late apoptosis of HepG2 cells: the early apoptotic cells increased from 5.18 ± 2.05% to 22.55 ± 1.94%, and the late apoptotic cells increased from 5.17 ± 1.65% to 15.53 ± 1.64%. These results confirmed that HWE can mediate early and late apoptosis of HepG2 cells.

### HWE Increased Cellular ROS Production

**FIGURE 7:**
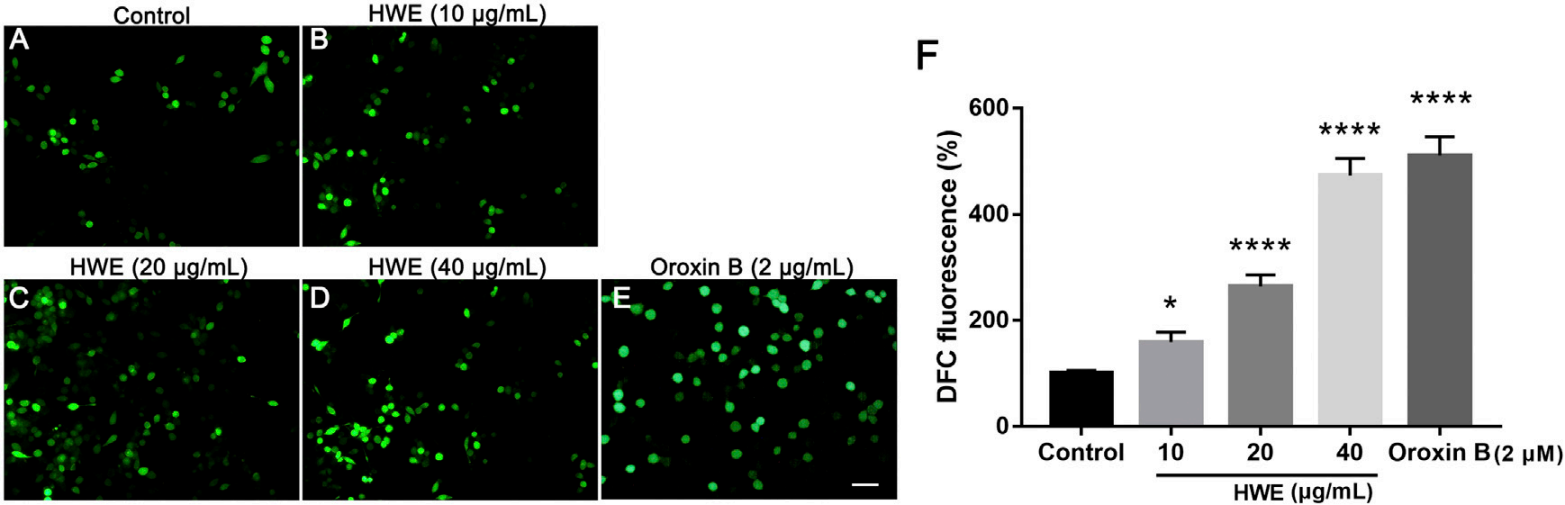
Intracellular ROS levels of HepG2 cells. **(A–D)** DCFH-DA staining of HepG2. cells with or without HWE treated, scale bar: 20 μm. **(E)** Quantitative analysis of DCFH-DA fluorescence intensity. The error bars represent SD. *p < 0.05 and ****p < 0.0001 vs control group.

To investigate the ROS production in HepG2 cells after HWE treatment, we performed DCFH-DA staining. As shown in Figures 7A–D, under the fluorescence microscope, the fluorescence intensity of cells treated with HWE increased significantly. As shown in Figure 7E, the levels of ROS in cells increased in a concentration-dependent manner after exposed to HWE which was similar to PTEN inhibitor Oroxin B. These results indicated that HWE can induce ROS production in HepG2 cells.

### HWE Increased the Expressions of Apoptosis-Related Proteins in HepG2 Cells

**FIGURE 8:**
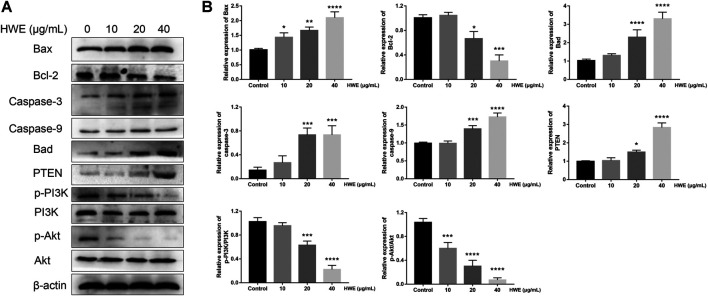
Effect of HWE on the expressions of mitochondrial apoptosis and PI3K pathway-related proteins. **(A)** Mitochondrial apoptosis and PI3K pathway-related proteins in HepG2 cells with or without HWE treated. **(B)** Quantitative analysis. The error bars represent the SD. *p < 0.05, **p < 0.01, ***p < 0.001 and ****p < 0.0001 vs control group.

Next, we further studied the regulatory effect of HWE on the apoptosis-related proteins in HepG2 cells. It should be noted that the expression of Bax was significantly increased by HWE, while the protein expression level of Bcl-2 was significantly decreased by HWE (Figures 8A,B). NIE can increase the protein expression of cytochrome c, Bad, caspase-9, and caspase-3 (Figures 8A,B).

### HWE Induced HepG2 Cells Apoptosis via PI3K-Akt-mTOR Pathway

To identify the mechanisms of pro-apoptosis by HWE, the expressions of PI3K and Akt were measured through Western blot assay. The phosphorylation of PI3K was decreased in HepG2 cells after exposure in HWE and the expression of the phosphorylation of Akt (Figures 8A,B). However, the expression of PTEN was significantly increased after treated with HWE. These results showed that HWE inhibited HepG2 cell proliferation may via the PTEN-PI3K-Akt signaling pathway.

## Discussion

In this study, we systematically analyzed the hub target and pathway of *Notopterygium incisum* through network pharmacology and predicted that HWE has an important impact on cancer pathways, especially Akt. Experimental validation demonstrated HWE has a significant inhibitory effect on HepG2 cell proliferation for the first time. These results indicated that HWE could induce apoptosis in HepG2 cells, increase the production of excessive ROS, and regulate the expression of apoptosis-related proteins in mitochondria. We speculated that the anti-hepatoma mechanism of HWE might through the PTEN-PI3K-Akt pathway.

In this study, a total of 154 compounds were identified using our drug prediction method. In the compound target network, compounds with high-degree may account for the cancer pathway and MAPK pathway. In this study, we systematically analyzed the hub target and pathway of *Notopterygium incisum* through network pharmacology, and the results showed that *Notopterygium incisum* had an important effect on Akt.

The PTEN-PI3K-Akt pathway is a very attractive target in the clinical trials of cancer treatment (Bartholomeusz and Gonzalez-Angulo, 2012). In normal cells, activation of the PTEN-PI3K-Akt signaling pathway is involved in cell proliferation, survival, and migration (Brazil et al., 2002). The abnormal activation of this pathway can promote the continuous growth of cancer cells (Dann et al., 2007). There is also evidence that the expression levels of mTOR and its downstream P70S6K in the PI3K-Akt-mTOR signaling pathway in HCC is significantly higher than in paracancerous and normal liver tissues (Li et al., 2014). Despite advances in drug research targeting the PI3K/Akt/mTOR pathway, there is still a clinical need for safer and more effective targeted drugs.

The PI3K/Akt/mTOR pathway plays a key role in tumor formation, metabolism, cell cycle progression, apoptosis, survival. Indeed, PI3K activity is very important for the retention of Bax in the cytoplasm, which regulates the degree of Bax translocation to mitochondria (Tsuruta et al., 2002). Apoptosis is the main route of programmed cell death, and it is a highly regulated and ordered cell destruction program, which helps to clear damaged or redundant cells and is an important target of cancer (Han et al., 2018). Apoptosis is mainly caused by the “exogenous” pathway mediated by death receptors and the “endogenous” pathway mediated by mitochondria, and these two pathways are closely related to the Bcl-2 family and mitochondrial proteins (Czabotar et al., 2014). The Bcl-2 family includes pro-life and pro-death-related proteins. It was generally accepted that the balance between the opposite proteins could regulate the sensitivity of cells to apoptosis (Volkmann et al., 2014). Our results confirmed that the expression of Bcl-2 was decreased and the level of Bax was increased by HWE in HepG2 cells, which induce apoptosis may be mediated by activation of the intrinsic apoptotic pathway. Further study found that HWE increased the activity of apoptosis-related protein in HepG2 cells, such as caspase-3, caspase-9, and cytochrome C.

Furthermore, we detected the expression level of PETN-PI3K-Akt in HepG2 cells. The expressions of p-PI3K and *p*-Akt were significantly reduced in HepG2 cells after exposure to HWE. The expression level of PETN was significantly up-regulated after treated with HWE. These results indicate that HWE regulates HepG2 cell apoptosis may via the PI3K-Akt-mTOR pathway.

## Conclusion

In summary, our results indicated that the HWE significantly inhibited the cell viability of HepG2. HWE can induce apoptosis in HepG2 cells, induce the production of a large number of ROS, and ultimately lead to apoptosis through the PTEN-PI3K-Akt pathway. This study provides a new choice for the drug development of liver cancer. A weakness of this study is that it is not known which component/multicomponent of HWE is the most important one responsible for the beneficial effect of the treatment of liver cancer.

## Data Availability

The original contributions presented in the study are included in the article/Supplementary Material, further inquiries can be directed to the corresponding author.
